# Reduced synaptic activity in neuronal networks derived from embryonic stem cells of murine Rett syndrome model

**DOI:** 10.3389/fncel.2014.00079

**Published:** 2014-03-26

**Authors:** Lydia Barth, Rosmarie Sütterlin, Markus Nenniger, Kaspar E. Vogt

**Affiliations:** Neurobiology and Pharmacology, Biozentrum, University of BaselBasel, Switzerland

**Keywords:** Rett syndrome, stem cell-derived neurons, neurodevelopment, electrophysiology, excitability, synaptic activity

## Abstract

Neurodevelopmental diseases such as the Rett syndrome (RTT) have received renewed attention, since the mechanisms involved may underlie a broad range of neuropsychiatric disorders such as schizophrenia and autism. In vertebrates early stages in the functional development of neurons and neuronal networks are difficult to study. Embryonic stem cell-derived neurons provide an easily accessible tool to investigate neuronal differentiation and early network formation. We used *in vitro* cultures of neurons derived from murine embryonic stem cells missing the methyl-CpG-binding protein 2 (*MECP2*) gene (MeCP2-/y) and from wild type cells of the corresponding background. Cultures were assessed using whole-cell patch-clamp electrophysiology and immunofluorescence. We studied the functional maturation of developing neurons and the activity of the synaptic connections they formed. Neurons exhibited minor differences in the developmental patterns for their intrinsic parameters, such as resting membrane potential and excitability; with the MeCP2-/y cells showing a slightly accelerated development, with shorter action potential half-widths at early stages. There was no difference in the early phase of synapse development, but as the cultures matured, significant deficits became apparent, particularly for inhibitory synaptic activity. MeCP2-/y embryonic stem cell-derived neuronal cultures show clear developmental deficits that match phenotypes observed in slice preparations and thus provide a compelling tool to further investigate the mechanisms behind RTT pathophysiology.

## INTRODUCTION

Rett (1966) described an unusual neurodevelopmental disorder in girls, now called Rett syndrome (RTT). Today the key diagnostic criteria for RTT are stereotypic hand movements, deficits in motor coordination, speech disorders, and autistic behavior (Hagberg and Hagberg, 1997). Children develop normally for 6–18 months after birth, reaching the usual motor, language and social milestones. This brief period of developmental progress is followed by stagnation with growth arrest and microcephaly. During the following rapid regression phase, the previously acquired skills are lost and a variety of neurological symptoms develop. These include sleep disturbances, problems with gait, decelerated head growth, breathing arrhythmia, stereotypical hand movements, loss of motor coordination, and seizures (Zoghbi, 2003; Moretti and Zoghbi, 2006; Chahrour and Zoghbi, 2007).

Amir et al. (1999) identified the primary cause of RTT as a defect in the *MECP2* gene on the X chromosome, coding for the methyl-CpG-protein 2 (MeCP2). More than 95% of individuals with classic RTT carry *de novo* mutations. MeCP2 is highly enriched in neurons in the central nervous system (Zhou et al., 2006). It regulates genes essential for neuronal survival, dendritic growth, synaptogenesis, and synaptic plasticity (Fukuda et al., 2005; Chang et al., 2006; Smrt et al., 2007). The function of MeCP2-targeted genes seems especially important in GABAergic neurons (Huang et al., 2007; Chao et al., 2010). GABAergic interneurons provide the main inhibitory function in the central nervous system and thereby contribute to the essential balance between excitation and inhibition. A disturbed excitation/inhibition balance will in severe cases result in epileptic discharges, which are found in 70–90% of RTT patients (Nissenkorn et al., 2010).

Generation of different mouse models in 2001 by targeting of the *MECP2* gene has provided significant advances in understanding of MeCP2 function and mimicking relevant aspects of human RTT (Chen et al., 2001; Guy et al., 2001; Shahbazian et al., 2002). MeCP2- null male mice (MeCP2-/y) were generated by replacing exons 3 and 4 of *MECP2* starting in early embryonic development (Guy et al., 2001). Most studies use such male hemizygous mice because they develop a severe and characteristic behavioral phenotype much earlier than female heterozygous mice. The mice develop motor impairments, tremor, breathing abnormalities, limb stereotypies, and epilepsy as in the human condition (Colic et al., 2013). Remarkably, it was later shown that re-expression of endogenous MeCP2 can reverse aspects of RTT in the adult (Guy et al., 2007). Within a few weeks the affected mice were largely indistinguishable from their wild type (wt) controls. This does not yet suggest a prompt therapeutic approach to RTT but it clearly establishes the principle of reversibility in this mouse model.

The causal link between MeCP2 dysfunction and the neurobehavioral phenotype is still unclear. Given the reversibility of the phenotype, a better understanding of the neuronal phenotype becomes more and more important. Especially early developmental stages are hard to study functionally since the neurons are not easily accessible for targeted manipulations at these stages.

Murine embryonic stem cell (mES)-derived neurons allow a straightforward functional analysis of neuron maturation from very early stages up to network formation.

Here we investigate the differentiation of neural precursors derived from MeCP2-/y mice and from the corresponding E14Tg2a wt background. We compared intrinsic parameters, such as resting membrane potential (RMP), the function of voltage-gated sodium- and potassium channels as well as generation of action potentials (APs) from immature and mature neurons. As soon as the neurons formed synaptic networks, we studied spontaneous excitatory and inhibitory synaptic activity and its maturation over time.

## METHODS AND MATERIALS

### CELL CULTURE AND DIFFERENTIATION

Embryonic stem cells derived from a E14Tg2a background with the MeCP2+/y (wt) and MeCP2-/y genotype were cultured and differentiated into neurons as described (Bibel et al., 2007). Briefly, after 4 days of embryoid body formation these are treated with 5 μM all-*trans*-retinoic acid (Sigma Inc., Buchs, Switzerland) for additional 4 days. Embryoid bodies are dissociated and neuronal precursors were plated on poly-L-ornithine (Sigma Inc., Buchs, Switzerland)/laminin (Roche Inc., Buchs, Switzerland) – coated glass cover slips (Assistent, Karl Hecht GmbH, Sondheim/Rhön, Germany). At day in vitro (DIV) 0 and 1 neuronal precursors were cultured in neural medium containing DMEM/F12, N-2 Supplement (100X) and penicillin/streptomycin and 1 mM glutamine (all Invitrogen Inc., Lucerne, Switzerland). From DIV 2 the medium was changed to the differentiation medium containing Neurobasal medium, B-27® Supplement (50X), N-2 Supplement (100X), 0.6 mM glutamine and penicillin/streptomycin (all Invitrogen Inc., Lucerne, Switzerland).

### ELECTROPHYSIOLOGY

Cover slips with neurons were transferred to a bath chamber mounted to an inverted microscope (Axiovert 25, Carl Zeiss GmbH, München, Germany). Experiments were performed on DIV 0–8 and DIV 11–23 neurons in culture using the whole-cell voltage-clamp technique. Data were collected using a Multiclamp 700A amplifier (Axon Instruments, Union City, CA, USA). We used electrodes with an open tip resistance of 4–5 MΩ obtained by pulling borosilicate pipettes (Clark, Warner Instruments Inc., Edenbridge, United Kingdom) with 1.5 mm external diameter and 1.17 mm internal diameter without filament to a tip diameter of ~1 *μ*m on a horizontal Puller (DMZ Puller, Zeitz GmbH, Martinsried, Germany). The intracellular solution was adapted to the medium the cells were cultivated in; for N2 medium it contained (mM): 110 K-D-gluconate, 5 KCl, 11 Tris-phosphocreatine, 1 EGTA, 4.5 MgATP, 10 HEPES, 0.3 Tris-GTP (pH 7.4 with KOH, 290 mOsm). The extracellular solution for cells coming from N2 medium used for DIV 0 and DIV 1 contained (in mM): 120 NaCl, 29 NaHCO_3_, 4 KCl, 1 CaCl_2_, 0.7 MgCl_2_, 18 glucose, pH 7.4 when bubbled continuously with 95% O_2_ and 5% CO_2_. Intracellular solution for cultures coming from complete medium contained (mM): 100 K-D-gluconate, 5 NaCl, 1 EGTA, 5 MgATP, 10 HEPES, and 0.5 Tris-GTP (pH 7.4 with KOH, 210 mOsm). The extracellular solution for complete medium contained (in mM): 125 NaCl, 26 NaHCO_3_, 1.25 NaH_2_PO_4_*H_2_O, 2.5 KCl, 1.0 MgSO_4_, 2.0 CaCl_2_ and 11 glucose, pH 7.4 when bubbled continuously with 95% O_2_ and 5% CO_2_. Voltage-gated sodium- and potassium channels were detected in voltage-clamp mode at a holding potential of -60 mV. The holding potential was changed in a stepwise fashion from -75 to +25 mV in 5 mV increments for 800 ms and the voltage-gated peak inward current and the sustained outward current (between 600–800 ms) were measured for each step. The inward currents were tetrodotoxin (TTX) sensitive, while the outward currents were blocked by tetraethyl-ammonium and 4-aminopyridine [TEA (3 mM) and 4-AP (1 mM; data not shown)]. For statistical comparisons the maximal evoked currents for a given cell were used. RMPs and APs were recorded in current-clamp mode. Somatic current injections were applied in 2.5 pA steps from -2.5 to +30 pA, in older cells up to +60 pA. Synaptic activity was measured in voltage-clamp mode using the same k-gluconate based internal in order to maximise cell-yield: to detect spontaneous excitatory synaptic currents (sEPSCs), cells were held at -60 mV, while for spontaneous inhibitory synaptic currents (sIPSCs), cells were held at -40 mV. This allowed us to unambiguously identify EPSCs and IPSCs in the same recording without pharmacological agents present, which might have affected network activity. Responses were filtered at 5 kHz and digitized at 20 kHz. The excitatory glutamate receptor blocker 2,3-dihydroxy-6-nitro-7-sulfamoyl-benzo[f]quinoxaline-2,3-dione (NBQX; 10 μM) and antagonists of inhibitory GABA_A_ receptors picrotoxin (100 μM) or bicuculline (20 μM) were added to the perfusate at the end of recordings to block the respective synaptic activity (Tocris Inc., Bristol, United Kingdom). All other chemicals were purchased from the same supplier (Sigma Inc., Buchs, Switzerland). Recorded sEPSC and sIPSC were detected and analyzed using Mini Analysis 6 (Synaptosoft, Decatur, Georgia). All other data analysis was done with IGOR PRO 6.0 (Wavemetrics, Lake Oswego, OR, USA) software. Two-way ANOVA were used for all statistical analysis (unless otherwise mentioned), with Tukey’s HSD tests where indicated.

### IMMUNOCYTOCHEMISTRY

Cells cultured on glass coverslips were rinsed twice with phosphate buffered saline (PBS) pH 7.4 and fixed with 10% neutral buffered formalin (Sigma Inc., Buchs, Switzerland) for 20 min at room temperature (RT). After rinsing with PBS, coverslips were permeabilized for 5 min in 0.2% TritonX-100/PBS, rinsed with PBS and incubated for 1 h at RT in a humidified chamber with the following primary antibodies and dilutions (rb: rabbit, ms: mouse): doublecortin (rb, 1:1000, Cell Signaling, Bio Concept, Allschwil, Switzerland), microtubule-associated protein 2 (MAP2; rb, 1:1000, Chemicon, Millipore Inc., Zug, Switzerland), glutamic acid decarboxylase, 67 kDa isoform, (GAD67; ms, 1:500, Chemicon, Millipore Inc., Zug, Switzerland) and synaptophysin (ms, 1:300, Sigma Inc., Buchs, Switzerland). After several washes with PBS, coverslips were incubated for 1 h with corresponding secondary antibody: Cy5 [donkey anti rabbit IgG (H+L), 1:300, Immuno Jackson, Suffolk, United Kingdom], Alexa 488 [donkey anti mouse IgG (H+L), 1:800, Invitrogen Inc., Lucerne, Switzerland], Alexa 488-phalloidin (1:400, Molecular Probes, Eugene, OR, USA) and 4′,6-diamidino-2-phenylindole (DAPI) (1:1000, Molecular Probes, Eugene, OR, USA). After several washes in PBS, coverslips were mounted in Mowiol-1188 as previously described (Baschong et al., 1999). Confocal sections were recorded with a confocal laser scanning microscope Leica TCS SPE with DMI 4000B (Leica Switzerland) and processed with Imaris software (Bitplane, Zurich, Switzerland) and Adobe Photoshop version 10.0 (Adobe Inc., San Jose, CA, USA).

## RESULTS

### MORPHOLOGICAL DEVELOPMENT

**FIGURE 1:**
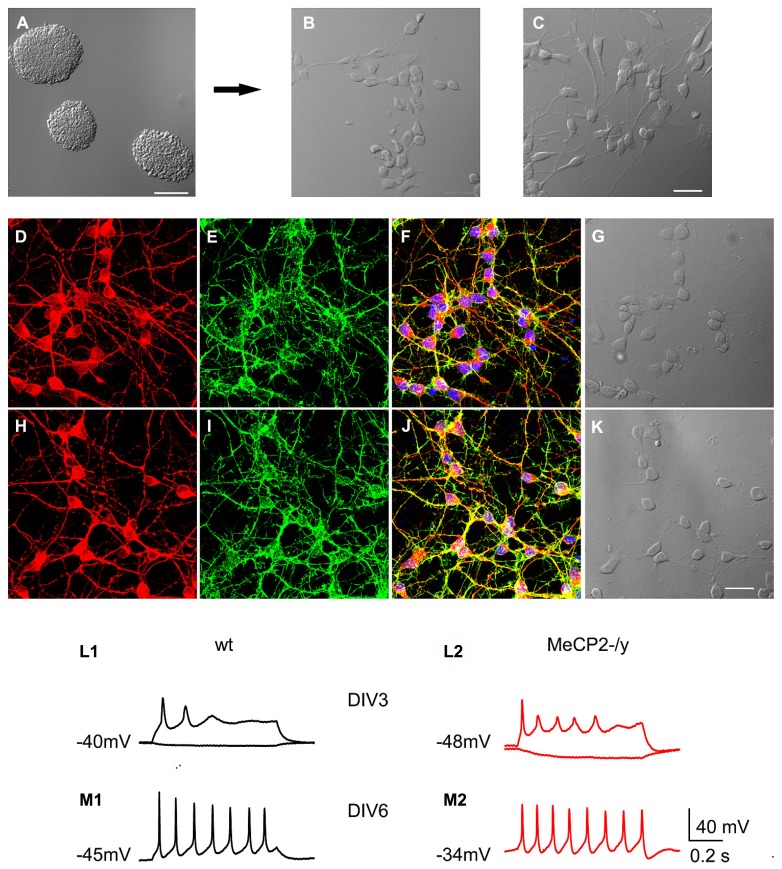
**mES cell-derived neurons show early maturation.** **(A–C)** DIC images of early neuronal differentiation in stem cell-derived cultures. **(A)** Image of embryoid bodies before dissociation; scale bar: 100 μm. Progenitor cells 6 h after plating **(B)** and at DIV 1 **(C)**; scale bar: 20 μm. Immunostainings against doublecortin **(D)**, actin staining with phalloidin **(E)**. **(F)** Overlay of doublecortin and phalloidin stain with DAPI stain (blue) and the corresponding DIC image **(G)** in wt cultures at DIV 3. **(H–K)** The corresponding pictures from MeCP2-/y cultures. Scale bar: 20 μm. **(L,M)** Early development of neuronal physiology; the numbers next to the traces indicate the resting membrane potential. **(L)** Reaction of wt and MeCP2-/y neurons to negative (bottom traces) and positive (top traces) somatic current injections at DIV 3. **(M)** Reaction of wt and MeCP2-/y neurons to positive somatic current injections at DIV 6.

We differentiated MeCP2+/y (wt) and MeCP2-/y mES cells from the same E14Tg2a background into neurons using an established protocol (Bibel et al., 2007). Progenitors dissociated from embryoid bodies (**Figure 1A**) showed already at DIV 0 a distinct, spindle shaped morphology (**Figure 1B**), which transformed into a multipolar shape by DIV 1 (**Figure 1C**). To characterize their morphological development, wt (**Figures 1D–F**) and MeCP2-/y cells (**Figures 1H–J**) were stained on DIV 3 with antibodies against doublecortin (**Figures 1D,H**), a marker of immature neurons. In addition we used the actin cytoskeleton label phalloidin (**Figures 1E,I**), the nuclear marker DAPI (**Figures 1F,J**) and differential interference contrast (DIC) images (**Figures 1G,K**). By DIV 3 more than 90% of the cells in culture were immature neurons with a multipolar shape. The DAPI staining indicated that the cell densities were similar in both genotypes (wt: 21.4 ± 2.2 cells/10000 μm^2^, *n* = 10; MeCP2-/y: 21.8 ± 3.0 cells/10000 μm^2^, *n* = 10, *p* > 0.91 Student’s *t*-test).

### FUNCTIONAL DEVELOPMENT OF VOLTAGE-GATED CURRENTS

The wt progenitors exhibited a RMP of -56 ± 2.2 mV at DIV 0 (*n* = 15) compared to -35 ± 3.0 mV for MeCP2-/y progenitors (*n* = 8). At DIV 2 RMP of wt cells (*n* = 21) depolarized to -41 ± 3.3 mV, while for MeCP2-/y (*n* = 16) we measured a RMP of -36 ± 2.1 mV. In the following days cells from both genotypes showed a gradual hyperpolarization to -57 ± 0.9 mV for wt and -57 ± 1.0 mV for MeCP2-/y, which stabilized around DIV 11 (*n* = 180 for wt and 151 for MeCP2-/y). Statistical analysis revealed a highly significant influence of developmental age (*p* < 0.01), but no significant effect of the genotype (*p* > 0.08) on the RMP.

**FIGURE 2:**
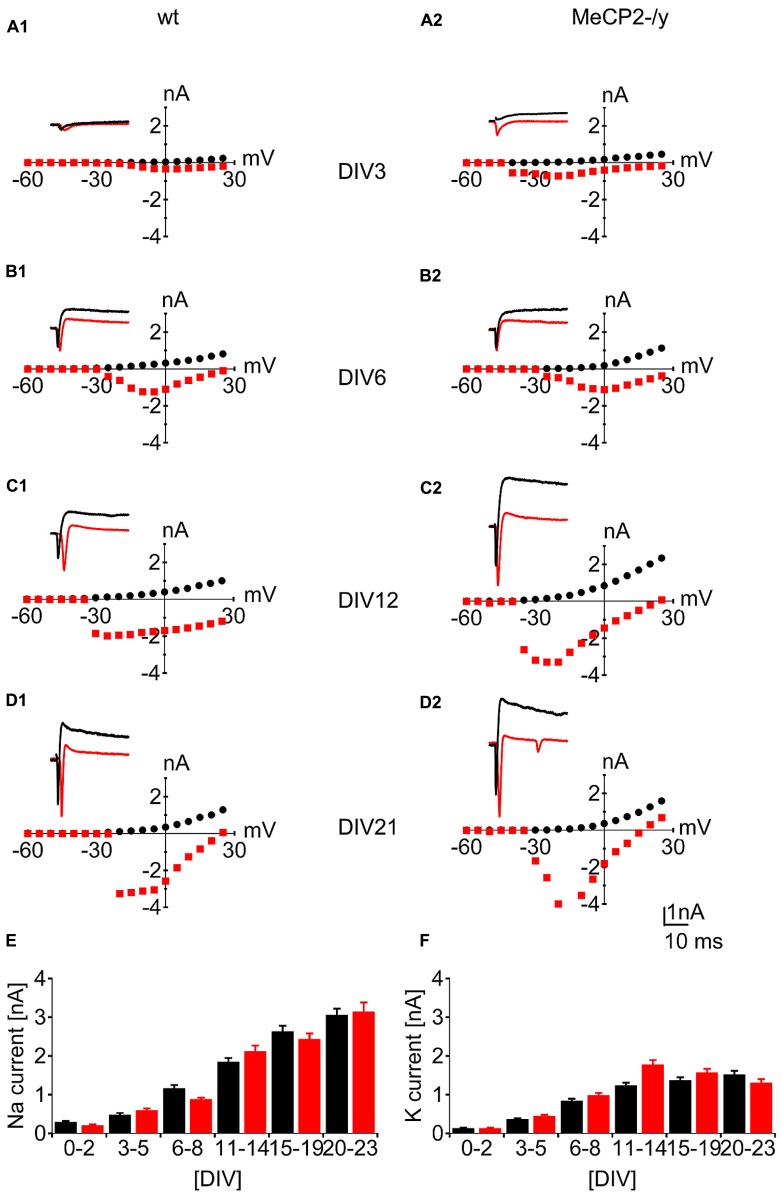
**Developmental characterization of voltage-gated sodium- and potassium currents.** Sample traces and I–V curves for voltage activated inward sodium currents (red) and voltage activated outward potassium currents (black). Data points for the I–V curves plot peak inward current (red squares) against the holding potential after depolarization and the sustained outward current (black dots) against the holding potential after depolarization. **(A–D)** Increase in voltage-activated whole-cell currents from DIV 3 to DIV 21. **(E)** Comparison of inward sodium current as a function of developmental age for wt (black) and MeCP2-/y (red) cultures. **(F)** Comparison of outward potassium currents as a function of developmental age for wt (black) and MeCP2-/y (red) cultures.

We observed voltage-dependent inward sodium currents (I_Na_) with fast activation and inactivation kinetics, as well as slow, non-inactivating outward potassium currents (I_K_) during all stages of differentiation. Already 6 h after plating in 6 of 10 cells a small I_Na_ of 0.04 ± 0.02 nA in wt and 0.02 ± 0.01 nA in MeCP2-/y and a I_K_ of 0.11 ± 0.03 nA in wt and 0.04 ± 0.01 nA in MeCP2-/y could be found. Both types of currents showed a substantial increase during development in culture (**Figure 2**). I_Na_increased continuously from 0.48 ± 0.04 nA in wt and 0.60 ± 0.04 nA in MeCP2-/y (DIV 3–5) to 3.06 ± 0.16 nA in wt and 3.14 ± 0.23 nA in MeCP2-/y (DIV 20–23; **Figure 2E**). For I_K_ an initial increase from 0.36 ± 0.025 nA in wt and 0.45 ± 0.03 nA in MeCP2-/y (DIV 3–5) to 1.24 ± 0.07 nA in wt and 1.78 ± 0.11 nA in MeCP2-/y (DIV 11–14) then leveled off at 1.52 ± 0.09 nA in wt and 1.31 ± 0.09 nA in MeCP2-/y (DIV 20–23; **Figure 2F**). For I_Na_ the effect of genotype was not significant (*p* > 0.82), while the developmental age exerted a highly significant effect (*p* < 0.01; *n* = 25 per day and genotype). Both genotype and developmental age significantly affected I_K_ (*p* < 0.01 for both variables, *n* = 25 per day and genotype). Pairwise *post hoc* analysis did not reveal a significant difference between genotypes at any specific time point (*p* > 0.5).

### ACTION POTENTIALS AND EXCITABILITY

**FIGURE 3:**
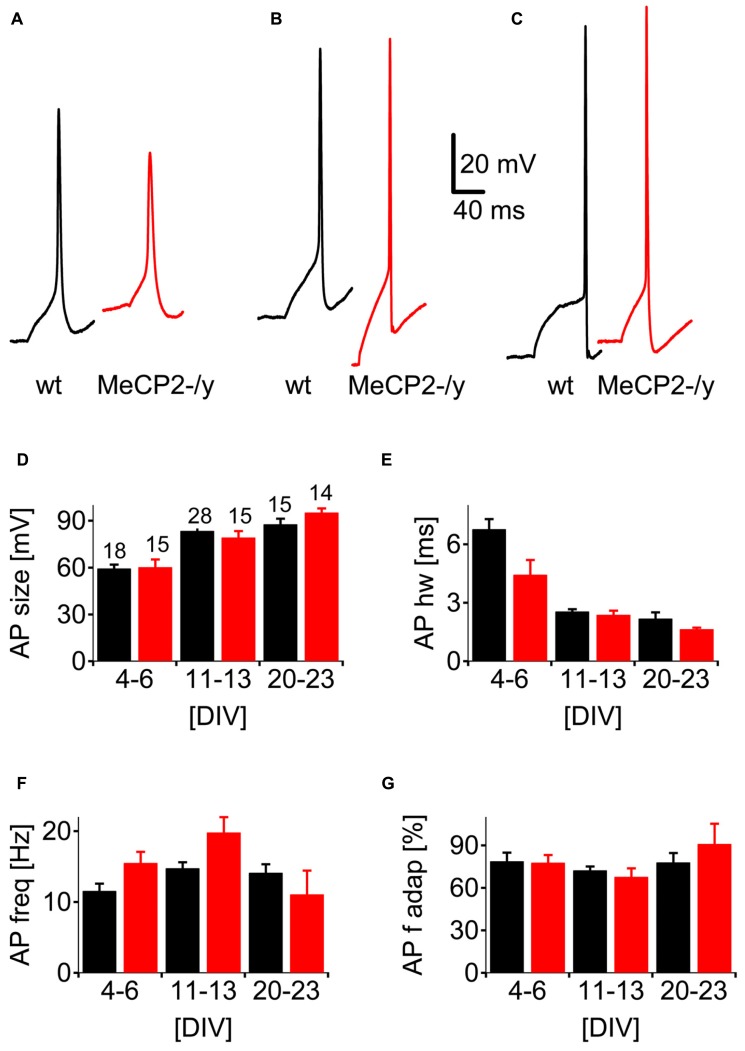
**Neuronal spiking patterns as a function of developmental age.** Representative single spikes evoked by supra-threshold current injections in cultured neurons at **(A)** DIV 6, **(B)** DIV 12, and **(C)** DIV 21. Repetitive spiking was evoked by current injections shown in **Figure 1M. (D–G)** Analysis of the spiking parameters in wt (black) and MeCP2-/y (red) cultures as a function of time in culture. **(D)** Data for the amplitude of the first AP, **(E)** the half-width at half height for the first AP, **(F)** the initial firing frequency and **(G)** the frequency at the end of the train relative to the initial firing frequency.

Action potentials were detected from DIV 3 (**Figure 1L**) in both genotypes. Typical APs are shown for DIV 6 (**Figure 3A**), DIV 12 (**Figure 3B**) and DIV 21 (**Figure 3C**). A quantitative analysis of the APs was performed between the age ranges DIV 4–6, DIV 11–13, and DIV 20–23 in 61 wt and 44 MeCP2-/y cells (**Figures 3D–G**) by measuring the size of the first AP in a train (see **Figure 1M**) and its half-width, as well as the initial frequency of APs in a train and their frequency adaptation. First AP size (**Figure 3D**) increased from DIV 4 to DIV 23 in wt neurons from 59.5 ± 2.5 to 87.6 ± 3.7 mV and in MeCP2-/y from 60.3 ± 4.9 to 95.7 ± 2.8 mV. First AP half width (**Figure 3E**) decreased over time in wt from 6.7 ± 0.5 to 2.2 ± 0.3 ms and in MeCP2-/y from 4.4 ± 0.7 to 1.6 ± 0.08 ms. Initial AP frequency within a train (**Figure 3F**) showed no clear developmental pattern and varied between 10–20 Hz for both genotypes. Cells from both genotypes exhibited a weak frequency adaptation to between 60 and 90% of the initial frequency (**Figure 3G**) throughout their development. Statistical analysis revealed a significant effect of age (*p* < 0.0.1), but not of genotype (*p* > 0.3) on AP size. Both age and genotype significantly affected the AP half-width (*p* < 0.01) with a significant interaction (*p* < 0.01). Initial AP frequency was significantly affected by both age (*p* < 0.01) and genotype (*p* < 0.05) without significant interaction (*p* > 0.2). AP frequency adaptation was significantly affected by age (*p* < 0.05), but not by genotype (*p* > 0.9).

### SYNAPTIC ACTIVITY

**FIGURE 4:**
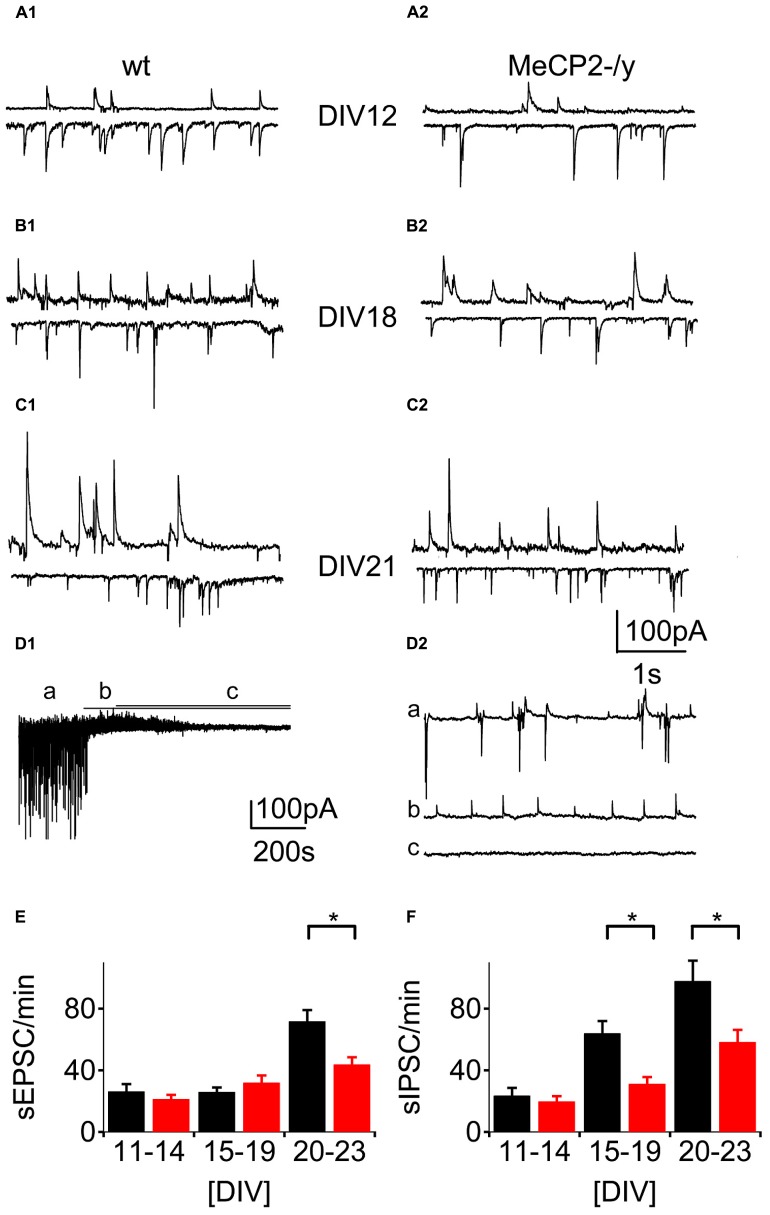
**Synaptic activity as a function of development.** **(A–C)** Traces of spontaneous activity in voltage-clamp. Top traces at -40 mV holding potential bottom traces at -60 mV, respectively. **(D)** Pharmacological characterization of synaptic currents. **(D1)** Sample trace of one experiment with baseline recording **(A)**, after bath application of NBQX (10 μM) and bath application of picrotoxin (100 μM; **C)**. **(D2)** Inserts show the same regions as in **(D1)** at higher temporal resolution. **(E)** Frequency of sEPSCs as a function of time in culture for wt (black) and MeCP2-/y (red) cultures. *p < 0.05. **(F)** Same data for sIPSCs. *p < 0.01.

We detected the first spontaneous excitatory (sEPSCs) and inhibitory (sIPSCs) post-synaptic currents by DIV 11 in neurons from both wt and MeCP2-/y cultures (**Figure 4**). We measured sEPSCs as inward currents (**Figures 4A–C** bottom traces) at a holding potential of -60 mV, while sIPSCs were best visible and measured as outward currents at a holding potential of -40 mV (**Figures 4A–C** top traces). sEPSCs were blocked by the addition of the AMPA-receptor blocker NBQX (10 μM) to the bath medium, whereas sIPSCs were blocked by bath application of the GABA_A_ receptor antagonists picrotoxin (100 μM) or bicuculline (20 μM) respectively, in all cultures tested (*n* = 21; **Figure 4D**).

Between DIV 11–23 the frequency of sEPSCs increased from 26.2 ± 4.8 to 71.8 ± 7.4 events per minute in wt (*n* = 186) and from 21.2 ± 2.6 to 43.8 ± 4.6 events per minute in MeCP2-/y (*n* = 172; **Figure 4E**). This represents a highly significant increase with age (*p* < 0.01) as well as a significant reduction at DIV 20–23 in excitatory activity in MeCP2-/y cultures compared to wt (*p* < 0.05).

The frequency of sIPSCs rose from 23.5 ± 5.1 to 97.8 ± 13.3 events per minute between DIV 11–23 for wt neurons, while in neurons from MeCP2-/y cultures it went from 19.7 ± 3.4 to 58.8 ± 7.9 events per minute over the same time (**Figure 4F**). Both developmental age (*p* < 0.01) and the cell’s genotypes (*p* < 0.01) exerted a highly significant influence in inhibitory synaptic activity.

**FIGURE 5:**
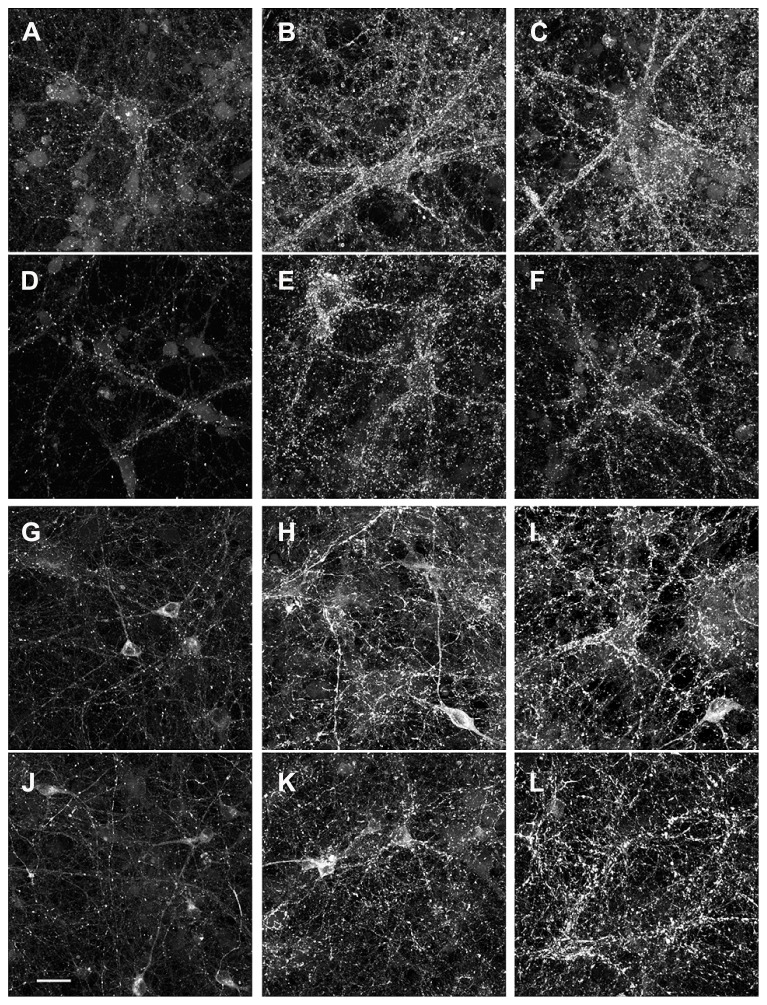
**Comparison of synaptic marker expression.** Mature neurons from wt **(A–C, G–I)** and MeCP2-/y **(D–F, J–L)** were assessed immunocytochemically on DIV 12, DIV 18 and DIV 21 for expression of the presynaptic marker synaptophysin **(A–F)** and the marker of GABAergic neurons GAD-67 **(G–L)**. Scale bar: 20 μm.

To see whether the reduced synaptic activity was accompanied by a change in synaptic density we stained cultures at DIV 12, 18, and 21 with antibodies against the neuronal marker MAP2 and against the presynaptic marker synaptophysin (**Figures 5A–F**). We measured synapse density by counting the number of synaptophysin positive puncta along 6–12 MAP2-positive dendrites per age range and genotype. In wt cultures the densities were 61 ± 2.2, 89 ± 8.9, and 64 ± 8.1 puncta/100 μm at DIV 12, 18, and 21 respectively. In MeCP2-/y cultures the values were 46 ± 2.6, 76 ± 7.3, and 81 ± 7.1 puncta/100 μm for the same time points. While age had a significant influence on the density of synaptophysin puncta (*p* < 0.01), there was no significant effect of the genotype (*p* > 0.45) on synapse density. We also stained the cultures against GAD67, a specific marker for GABAergic neurons. A clear increase in staining could be observed from DIV 12 to 18 to 21, without an obvious difference in the amount of GAD67 immunoreactivity between the two genotypes (**Figures 5G–L**). Between DIV 18–21 the percentage of GAD67- and MAP2-double-positive neurons among all MAP2-positive cells was 41+/-3 % for wt and 36+/-2 % for MeCP2 -/y cultures (*n* = 8 for each genotype, *p* > 0.39, *t*-test).

## DISCUSSION

Networks formed by neurons differentiated from MeCP2-/y mES cells show clear functional deficits compared to wt cultures. Both overall excitatory- and particularly inhibitory synaptic activity was clearly significantly lower in MeCP2-/y compared to wt cultures. Both sEPSCs and sIPSCs appeared at the same age (DIV 11) in MeCP2-/y and wt cultures; and while we could not statistically resolve it, the deficit in synaptic activity appears larger at later compared to early developmental stages. We did not detect a significant difference in the synapse density between wt and MeCP2-/y cultures. A deficit in initial synapse formation or later in the number of synapses can therefore not fully explain the reduced synaptic activity. While we did not observe substantial differences in cell densities between MeCP2-/y and wt cultures during development, we cannot rule out subtle differences in cell numbers, which may contribute to our findings. In particular, small changes in the number of interneurons can also not be ruled out as a reason for some of the deficit in GABAergic signaling, even though our GAD67 stains did not show overt differences between the two genotypes. Due to the technical difficulties in unambiguously differentiating between glutamatergic and GABAergic neurons we pooled the recordings from all recorded cells. Since glutamatergic cells are clearly dominant in our cultures, our findings certainly apply to this cell type. For future studies it would be interesting to look at synaptic activity in the different neuron types and to further investigate the mechanisms underlying the deficit in synaptic activity.

We found small, but significant differences in voltage-gated potassium currents and in AP half-with and -frequency between genotypes. The biological significance of these small differences is not clear. Voltage-gated potassium currents are significantly stronger in young MeCP2-/y neurons compared to age-matched wt neurons; this is compatible with the shorter AP half-width that we observe in young MeCP2 deficient neurons. The AP frequency is significantly higher in MeCP2-/y cultures; however, the effect is not stable over time. Since MeCP2-/y neurons actually show a higher propensity to spike and since the effects on synaptic activity are most pronounced at later developmental stages the lower synaptic activity is clearly not a consequence of lower excitability in MeCP2-/y cultures.

Previous studies using ES and iPS cultures have found differences in the activity of voltage-sensitive sodium channels in cultures of MeCP2-/y neurons (Okabe et al., 2010; Farra et al., 2012). Such differences were not found in another study of cultured neurons (Marchetto et al., 2010). Interestingly we do not find a difference in I_Na_, but in I_K_ between MeCP2-/y and wt cultures in our system. Several recent studies have looked at the impact of MeCP2 mutations on intrinsic excitability in different brain regions. In one study on locus coeruleus neurons, the authors found altered expression levels of several voltage-dependent conductances in MeCP2-/y mice compared to wt controls (Zhang et al., 2010a). APs had a lower threshold, but were slightly prolonged in MeCP2-/y neurons; the overall effect of the loss of MeCP2 on neuronal excitability in this study depended somewhat on the cell type studied (Zhang et al., 2010a). In other brain regions, such as the cortex, no differences in intrinsic excitability were detected between MeCP2 deficient and wt mice (Dani et al., 2005). MeCP2 expression seems to affect neuronal excitability relatively mildly and in a cell-type dependent manner.

Several groups have studied synapse development and plasticiy in MeCP2 mutated mice (Nelson et al., 2006; Chao et al., 2007; Dani and Nelson, 2009; Wood et al., 2009; Zhang et al., 2010b). In all of these studies, deficits in synaptic maturation either of excitatory, or inhibitory connections were found after a certain developmental delay. This indicates that deficits in connectivity after an initially normal period of synapse formation are a common finding in mouse models of RTT. A lack of fundamental deficits in neuronal excitability, paired with a deficit in synapse maturation is also compatible with RTT patient’s symptoms, which occur after a phase of initially normal mental development. We now describe a variant of such a pathology in our mES cell-derived neurons.

The molecular nature of the synaptic deficit has not yet been elucidated for any of the systems tested. The initially normal development and the different effects on either excitatory or inhibitory synaptic transmission in various brain regions argue against key elements of the synaptic release machinery to be affected. In some studies a reduced number of dendritic spines or of synapses was described (Fukuda et al., 2005; Smrt et al., 2007). While we found no difference in the overall density of presynaptic terminals, we cannot be sure that the exact distribution of terminals is the same in MeCP2-/y compared to wt cultures. Different studies have found different types of synapses to be affected by a lack of MeCP2, depending on the brain region studied (Noutel et al., 2011; Durand et al., 2012). Alterations in cell adhesion molecules or scaffolding proteins with differential distributions in different brain regions could explain such findings.

Murine embryonic stem cell cell-derived neuronal cultures can produce relatively homogeneous neuronal tissue with an essentially limitless supply. Transcriptome analysis of such tissue might yield candidate molecules responsible for the deficits we observe. Key elements of the known RTT pathology in early developing neural networks could be replicated in our system. This makes it a useful tool to further investigate functional deficits of MeCP2 deficient neurons and to study candidate interventions aimed at slowing or reversing the changes observed.

## AUTHOR CONTRIBUTIONS

Lydia Barth: conception and design, data collection, analysis and interpretation, manuscript preparation. Rosmarie Sütterlin: conception and design, data collection, analysis and interpretation. Markus Nenniger: data analysis and interpretation. Kaspar E. Vogt: conception and design, data analysis and interpretation, manuscript preparation.

## Conflict of Interest Statement

The authors declare that the research was conducted in the absence of any commercial or financial relationships that could be construed as a potential conflict of interest.
